# Theranostics of Bone Metastases: The Role and Prospects of Bisphosphonate Radiopharmaceuticals

**DOI:** 10.3390/ph19020295

**Published:** 2026-02-10

**Authors:** Yu Qian, Guangxing Yin, Yuhao Jiang, Peiwen Han, Junbo Zhang

**Affiliations:** 1 Key Laboratory of Radiopharmaceuticals of Ministry of Education, NMPA Key Laboratory for Research and Evaluation of Radiopharmaceuticals (National Medical Products Administration), College of Chemistry, Beijing Normal University, Beijing 100875, China; 202421150122@mail.bnu.edu.cn (Y.Q.); 202331150067@mail.bnu.edu.cn (G.Y.); jiangyuhao0838@163.com (Y.J.); 202431150080@mail.bnu.edu.cn (P.H.); 2 Key Laboratory of Beam Technology of the Ministry of Education, School of Physics and Astronomy, Beijing Normal University, Beijing 100875, China

**Keywords:** bone metastasis, bisphosphonates, bone imaging, theranostics, radiopharmaceutical

## Abstract

Bone metastasis is among the most common complications of advanced malignant tumors and severely affects prognosis in patients. Nuclear medicine, particularly bone-targeted radiopharmaceuticals, plays a unique and pivotal role in the diagnosis and treatment of bone metastases. This review systematically outlines the evolutionary trajectory of bone-targeted radiopharmaceuticals. It revisits functional bone imaging agents based on Single Photon Emission Computed Tomography (SPECT) and Positron Emission Tomography (PET), as well as recently developed therapeutic radiopharmaceuticals for bone metastases. Building on this foundation, this article focuses on the advanced paradigm of “theranostics” in nuclear medicine, encompassing strategies for theranostic radionuclide pairing and the development of single-radionuclide theranostic agents, aiming to achieve individualized and precise dosimetry. Moreover, this review emphasizes bone-targeting molecular scaffolds, such as bisphosphonates, and highlights their potential and direction for optimization through rational drug design, with the goal of developing a new generation of highly effective and low-toxicity theranostic platforms. This work aims to provide systematic insights for enhancing the precise management of bone metastases.

## 1. Introduction

Bone is among the most common sites of distant metastasis in many malignant tumors, such as lung cancer, breast cancer, and prostate cancer [1,2,3]. Cancer cells from the primary site acquire invasive capabilities through epithelial–mesenchymal transition, enter the bloodstream, evade immune clearance via various mechanisms, and ultimately “home” to the unique immunosuppressive microenvironment of the bone marrow. The abundant regulatory T cells, myeloid-derived suppressor cells, and other components within the bone marrow provide a natural sanctuary for cancer cells, allowing them to colonize and even remain dormant for extended periods. Once “awakened” by signals such as locally released growth factors, cancer cells interact with the bone microenvironment, establishing a self-reinforcing “vicious cycle” that leads to the formation and progression of bone metastases [1,4,5,6,7]. This process not only causes intractable bone pain but also may induce skeletal-related events such as pathological fractures and spinal cord compression, severely impairing the quality of life of patients and prognosis [2,3,8,9].

The abnormal bone metabolic activity accompanying the bone metastasis process provides important information for early diagnosis and intervention [10,11,12]. Nuclear medicine plays an irreplaceable role in this context, leveraging its unique advantages of targeted delivery, functional imaging, and internal radiation therapy as an integrated approach [13,14,15]. Taking [^99m^Tc]Tc-MDP, which has been widely used clinically for decades, as an example, this drug achieves targeted distribution through the high affinity of its phosphonate groups for hydroxyapatite in bone. Combined with SPECT imaging, it enables systematic screening of the entire skeleton in a single session, offering significant value in detecting multiple and occult metastatic lesions [16,17,18]. Its imaging basis lies in changes in bone metabolic activity, allowing it to capture early signals of reactive bone remodeling stimulated by tumors before significant structural bone destruction occurs. It often indicates the presence of bone metastases earlier than X-ray plain films do [19,20,21].

**Figure 1:**
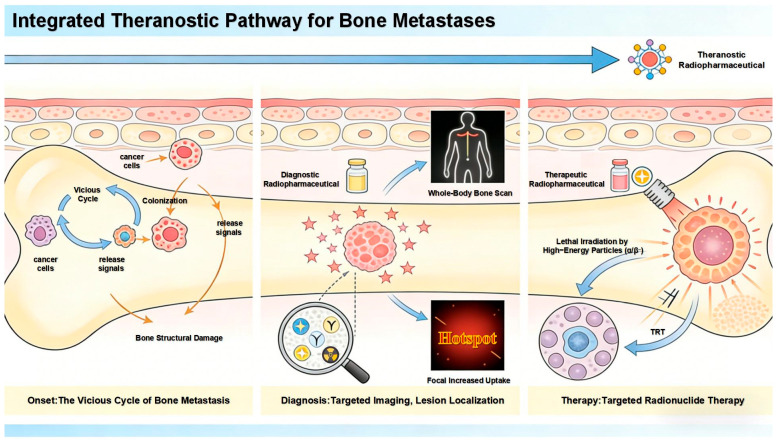
Schematic Diagram of the Integrated Theranostic Pathway for Bone Metastases.

Building on confirmed diagnoses, nuclear medicine further provides targeted treatment options [22,23,24,25,26,27,28]. For instance, [^223^Ra]RaCl_2_, as an alpha particle emitter, is selectively deposited on metabolically active bone surfaces at metastatic sites by following a metabolic pathway similar to that of calcium, achieving precise internal radiation therapy [29]. It has demonstrated clear efficacy in alleviating bone pain and prolonging survival [30,31]. The diagnostic and treatment pathway for bone metastasis is shown in Figure 1.

However, clinical needs and technological development never cease. Current research frontiers are dedicated to advancing on the classic foundation, with the core direction focused on advancing “theranostics” to a higher level. The ideal model involves constructing theranostic pairs based on the same molecular scaffold (e.g., using the same targeting molecule conjugated with diagnostic and therapeutic radionuclides) or developing radiopharmaceuticals in which a single radionuclide possesses both diagnostic and therapeutic properties [32,33,34,35,36]. This ensures that the imaging information obtained during the diagnostic phase can perfectly predict the in vivo distribution and retention of the therapeutic agent, thereby paving the way for truly individualized, precise dosimetry that achieves the specific treatment of what is visualized.

In this evolutionary process, bisphosphonates, as classic bone-targeting molecules, continue to exhibit renewed vitality because of their stable and strong affinity for hydroxyapatite [15,37,38,39,40,41,42]. Researchers are committed to rationally designing and modifying their chemical scaffolds and integrating them as targeting modules into the construction of novel radiopharmaceutical probes. The goal is to develop a new generation of bone metastasis theranostic platforms with greater specificity, improved therapeutic indices, and the ability to truly achieve a closed-loop theranostic cycle.

This article aims to systematically review the evolutionary path of bone-targeted radiopharmaceuticals, with a particular focus on their theranostic applications based on bisphosphonate-targeting molecules (featuring a P-C-P backbone). The full text first elaborates on bone imaging agents based on SPECT and PET, as well as therapeutic radiopharmaceuticals based on β^−^, α, and Auger electrons, analyzing their mechanisms, advantages, and limitations. It then delves into the strategies for achieving theranostics, including matched radionuclide pairs and single-radionuclide theranostic platforms. Finally, directions for the optimization and clinical translation of targeting scaffolds represented by bisphosphonates in next-generation theranostic drugs are anticipated, with the goal of providing systematic insights for the precise nuclear medicine management of bone metastases.

## 2. Bisphosphonate-Based Imaging Agents for Bone

On the basis of the imaging modality, bisphosphonate-based bone imaging agents can be primarily classified into two categories: those for Single Photon Emission Computed Tomography (SPECT) tracers and those for Positron Emission Tomography (PET) tracers.

### 2.1. SPECT Tracers

Technetium-99m (^99m^Tc) is among the most widely used radionuclides in nuclear medicine for SPECT owing to its excellent physical and chemical properties [43].

From a physical standpoint, ^99m^Tc is ideal for SPECT imaging. It emits a 140 keV gamma ray, which offers an optimal balance between tissue penetration and collimation for high-resolution images. Its 6 h half-life permits sufficient radiation for diagnosis while minimizing the patient dose. Furthermore, its on-demand availability via the ^99^Mo/^99m^Tc generator allows convenient transportation and instant labeling. Chemically, technetium exists in multiple oxidation states. The stable pertechnetate (TcO_4_^−^, +7) is reduced to lower states (+1, +3, +4 or +5), forming complexes with ligands via donor atoms (e.g., O, N, S, P and C such as in Tc(CO)x, Tc(CN)x, and Tc(aryl) compounds). This enables ^99m^Tc to be labeled onto bioactive molecules for targeted imaging.

The phosphonate groups (-PO_3_H_2_) in the molecular structure of bisphosphonates are rich in highly electronegative oxygen atoms, which serve as excellent electron donors capable of coordinating with metal ions. This key chemical characteristic enables the ^99m^Tc nuclide to coordinate directly with bisphosphonates without the need for any intermediary linkers, resulting in the formation of a classic direct labeling strategy. Technetium-99m-labeled methylene diphosphonate ([^99m^Tc]Tc-MDP) is the most successful example of this approach and has been used clinically as the gold standard for bone imaging for many years [16,17,18,44].

However, [^99m^Tc]Tc-MDP also has limitations, with the most notable being its relatively slow clearance rate from soft tissues. Imaging is typically performed 2–4 h after injection to ensure sufficient bone-to-background contrast [45]. Despite this drawback, its successful direct labeling paradigm has provided a core concept and template for subsequent development. On the basis of this strategy, researchers have derived a series of ^99m^Tc-labeled bisphosphonate compounds, including alendronate, ibandronate, risedronate, and others [46,47,48,49].

**Figure 2:**
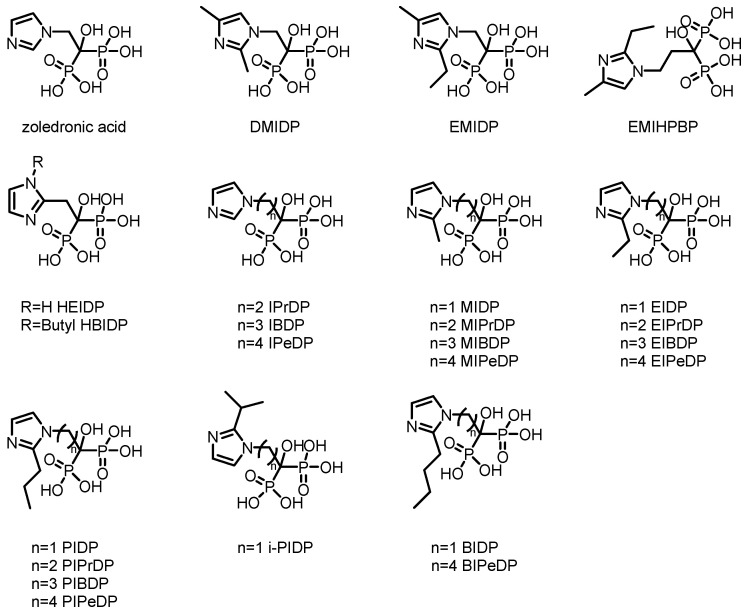
The chemical structures of zoledronic acid and some of its derivatives.

Furthermore, using zoledronic acid as a lead compound, researchers have systematically modified and optimized its structure, developing a range of novel derivatives with potentially improved properties. The chemical structure of zoledronic acid analogs is shown in Figure 2, and the relevant data of corresponding markers are presented in Table 1.

In the direct labeling method, the phosphate group serves the dual function of both coordinating with ^99m^Tc and targeting bone recognition, which may reduce its affinity for bone tissue. Therefore, introducing a specialized chelating group to separate the coordination function from the targeting recognition is highly important for improving the bone affinity of ^99m^Tc-labeled compounds.

**Figure 3:**
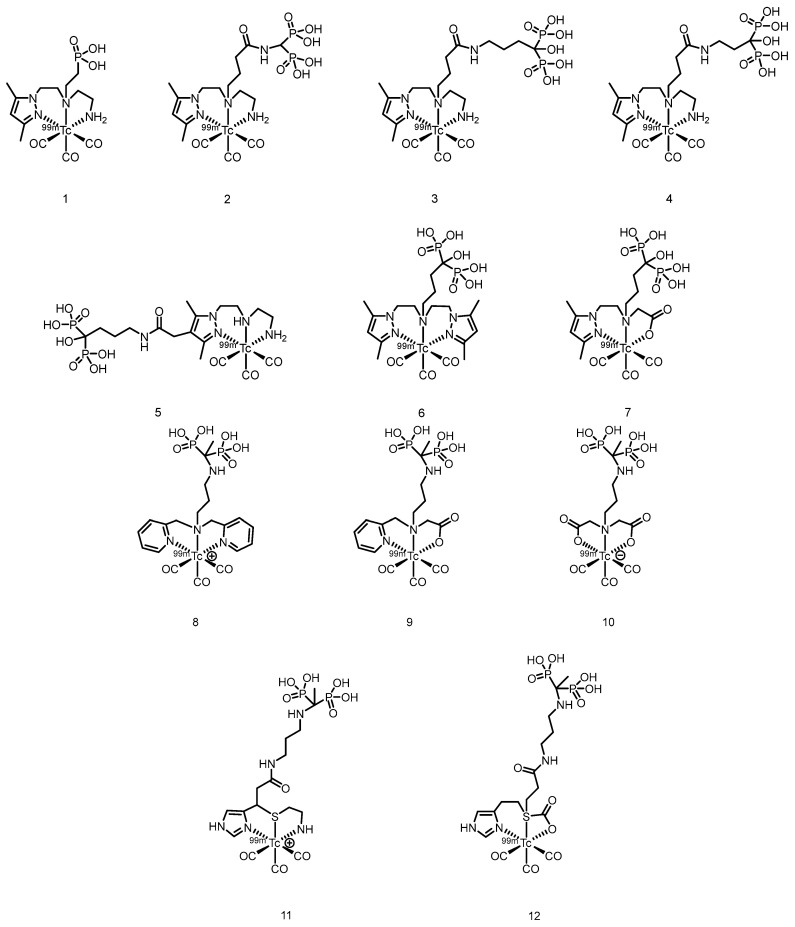
The possible structures of phosphate-targeting tricarbonyl technetium complexes.

In 2007, Palma et al. [64] developed ^99m^Tc(I)-tricarbonyl complexes using a pyrazolyl-diamine chelator, including monophosphonate-modified [^99m^Tc(CO)_3_(pz-MPOH)]^+^ (**1**) and bisphosphonate-conjugated [^99m^Tc(CO)_3_(pz-BPOH)]^+^ (**2**). (The proposed structures of Compound **1** and Compound **2** are shown in Figure 3). All the complexes were obtained as single species with >95% RCP. Complex 2 demonstrated excellent in vitro/in vivo stability, rapid blood clearance, and predominantly renal excretion. It showed moderate bone uptake (3.04 ± 0.47% ID/g at 4 h) with a favorable bone-to-background ratio, although it was significantly lower than that of clinical [^99m^Tc]Tc-MDP. This work validated the use of a pyrazolyl-diamine/tricarbonyl platform for the development of well-defined bone-seeking agents. Liu et al. [65] successfully prepared ^99m^Tc(CO)_3_-ABP by combining the fac-[^99m^Tc(CO)_3_(H_2_O)_3_]^+^ precursor with alendronate and systematically compared it with conventionally labeled ^99m^Tc-ABP. The tricarbonyl complex exhibited an RCP exceeding 90% and remained stable for 6 h at room temperature. Its partition coefficient (log *P* = −0.76) indicated weak hydrophilicity, which was significantly lower than the strong hydrophilicity of ^99m^Tc-ABP (log *P* = −3.39). Biodistribution studies revealed that although ^99m^Tc(CO)_3_-ABP achieved a bone uptake of 9.53% ID/g at 180 min, it was significantly lower than that of ^99m^Tc-ABP. Concurrently, the complex showed high retention in the liver, kidneys, lungs, and blood, indicating that its primary metabolic pathway involves hepatic and renal clearance. Compared with ^99m^Tc-ABP, ^99m^Tc(CO)_3_-ABP had substantially lower key targeting ratios, such as bone-to-liver and bone-to-blood ratios. In 2011, Palma et al. [66] developed two novel ^99m^Tc(I)-tricarbonyl bisphosphonate complexes, [^99m^Tc(CO)_3_(pz-ALN)]^+^ (**3**) and [^99m^Tc(CO)_3_(pz-PAM)]^+^ (**4**). (The proposed structures of Compound **3** and Compound **4** are shown in Figure 3). These complexes utilize a pyrazolyl-containing backbone (pz) as the chelating unit, conjugated with pamidronate and alendronate, respectively, for bone targeting. Both complexes showed high RCP (>95%), good hydrophilicity (log *P*_o/ω_ values of −2.00 ± 0.02 and −1.94 ± 0.02) and excellent hydroxyapatite binding comparable to that of [^99m^Tc]Tc-MDP. Biodistribution studies in mice revealed that compounds **3** and **4** achieved high bone uptake similar to that of [^99m^Tc]Tc-MDP (18.3 ± 0.6% ID/g and 17.3 ± 6.1% ID/g at 1 h postinjection), along with a higher total radioactive excretion rate (66.8% ID and 65.6% ID at 1 h postinjection), efficient clearance from blood and soft tissues, and superior bone-to-blood and bone-to-muscle ratios at 4 h postinjection compared with those of [^99m^Tc]Tc-MDP. Whole-body gamma camera imaging in rats further confirmed the promising potential of compounds **3** and **4** as bone imaging agents. In 2013, Fernandes et al. [67] further modified the linker moiety and designed and synthesized three ligands suitable for tricarbonyl technetium labeling, obtaining the corresponding compounds **5** to **7**. (The proposed structures of Compounds **5** to **7** are shown in Figure 3). Among them, compound **5** was obtained via radiosynthesis, with both radiochemical yield and purity exceeding 95%. In vitro studies revealed that the binding of compound **5** to hydroxyapatite was comparable to that of the [^99m^Tc]Tc-MDP and remained stable without degradation after 24 h of incubation in PBS and human serum. In BALB/c mouse models, compound **5** demonstrated rapid bone uptake (17.1 ± 3.6% IA/g in the femur at 1 h postinjection) and fast blood clearance, with bone-to-blood and bone-to-muscle radioactivity ratios significantly greater than those of [^99m^Tc]Tc-MDP. In MDA-MB-231 breast cancer cells, the cellular uptake of compound **5** reached 4–6% after 6 h, with 35% of the accumulated activity localized in the cytoplasm—substantially higher than that of other complexes in the same series. However, this series of complexes showed notable hepatic radioactive uptake in mice. In 2016, Makris et al. [68] reported five tricarbonyl technetium bisphosphonate compounds, namely compounds **8**–**12**. (The proposed structures of Compounds **8** to **12** are shown in Figure 3). Among them, compounds **8**–**11** were obtained with RCY exceeding 90% and, despite being hydrophilic, exhibited good in vitro stability. In biodistribution studies in BALB/c mice, all ^99m^Tc complexes demonstrated high bone uptake and rapid tissue clearance. Notably, compound **8** showed significantly higher bone uptake at 1 h post-injection than [^99m^Tc]Tc-MDP (19.44 ± 1.44 % ID/g vs. 13.18 ± 1.96 % ID/g). Moreover, its bone-to-blood ratios were significantly superior both at 1 h and 4 h after administration. The compounds were mainly excreted renally, although high kidney uptake was also observed.

**Figure 4:**
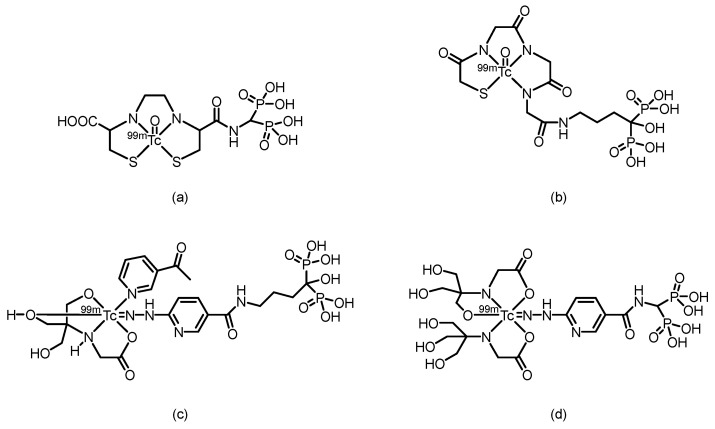
The possible structures of ^99m^Tc-complex-conjugated bisphosphonate compounds: (**a**) ^99m^Tc-EC-AMDP, (**b**) ^99m^Tc-MAG3-HBP, (**c**) ^99m^Tc-HYNIC-HBP, and (**d**) ^99m^Tc-HYNIC-AMDP.

In 2002, Verbeke et al. [69] synthesized ^99m^Tc-EC-AMDP (its proposed structure is shown in Figure 4), a compound obtained by covalently conjugating the renal imaging agent ^99m^Tc-ethylene dicysteine (^99m^Tc-EC) with aminomethylenediphosphonic acid (AMDP), via a seven-step reaction. The conjugate could be efficiently labeled with ^99m^Tc at a pH ≥ 12 and room temperature. HPLC analysis confirmed that the labeled product consists of two major components, with reproducible relative ratios and good stability over time. In biodistribution studies in rats, compared with [^99m^Tc]Tc-MDP, the new compound resulted in similar bone uptake at 30 and 60 min but significantly greater bone uptake at 120 min postinjection. Furthermore, residual blood activity was lower at all time points, resulting in significantly higher femur-to-blood activity ratios. In imaging studies in rabbits and a baboon, high-quality bone scans with clear skeletal visualization and low soft-tissue activity were obtained at 1 h and 2 h after injection, respectively. The plasma clearance rate of the compound was slightly faster than that of the [^99m^Tc]Tc-MDP in the baboon. However, the compound still has certain limitations: the total preparation may contain components with lower affinity for bone, and in the baboon model, it did not demonstrate a significant performance improvement compared with that of existing ^99m^Tc-diphosphonate imaging agents.

In 2006, Ogawa et al. [70] reported two ^99m^Tc-chelate–bisphosphonate conjugates (^99m^Tc-MAG3-HBP and ^99m^Tc-HYNIC-HBP). (Their proposed structures are shown in Figure 4). Alendronate was conjugated to mercaptoacetyltriglycine (MAG3) and hydrazinonicotinamide (HYNIC), successfully yielding two ligands (MAG3-HBP and HYNIC-HBP), which were then radiolabeled with ^99m^Tc. After purification, both ^99m^Tc-MAG3-HBP and ^99m^Tc-HYNIC-HBP (using tricine and 3-acetylpyridine as coligands) exhibited radiochemical purities greater than 95%. In vitro hydroxyapatite binding assays demonstrated that ^99m^Tc-HYNIC-HBP had a greater affinity for bone than the clinically used [^99m^Tc]Tc-HMDP [16,71] did. Biodistribution studies in rats revealed that compared with [^99m^Tc]Tc-HMDP, ^99m^Tc-HYNIC-HBP achieved greater bone uptake, with comparable blood clearance rates. Unlike ^99m^Tc-MAG3-HBP, which exhibited delayed blood clearance because of high serum protein binding (97.7%), ^99m^Tc-HYNIC-HBP showed lower serum protein binding (88.7%). Consequently, compared with [^99m^Tc]Tc-HMDP, ^99m^Tc-HYNIC-HBP resulted in a significantly higher bone-to-blood ratio. These results indicate that ^99m^Tc-HYNIC-HBP, designed on the basis of the concept of a bifunctional radiopharmaceutical, has potential as a bone imaging agent. In 2011, Liu et al. [72] synthesized a conjugate of HYNIC with aminomethylenediphosphonic acid (AMDP), which was subsequently labeled with ^99m^Tc using tricine as a coligand to form the ^99m^Tc-HYNIC-AMDP complex (its proposed structure is shown in Figure 4). This complex exhibited high RCP (>95%), good hydrophilicity (log *P* = −2.77), and excellent stability in vitro. Biodistribution studies in normal ICR mice revealed that ^99m^Tc-HYNIC-AMDP exhibited high bone uptake (35.08% ID/g at 1 h) and high bone-to-blood and bone-to-muscle ratios. Furthermore, it showed superior bone-targeting properties and faster blood clearance than conventional [^99m^Tc]Tc-MDP did at early time points, indicating its potential value for early-phase bone imaging.

**Figure 5:**
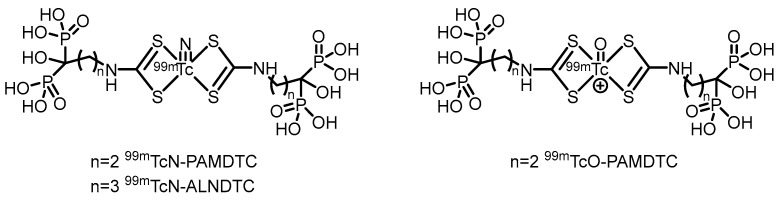
The possible structures of ^99m^Tc-complex-conjugated bisphosphonate compounds: ^99m^TcN-PAMDTC, ^99m^TcN-ALNDTC, ^99m^TcO-PAMDTC.

In 2017, our group [73] reported a novel alendronate dithiocarbamate (ALNDTC), which was radiolabeled with the ^99m^TcN core via a ligand exchange reaction to form the ^99m^TcN-ALNDTC complex (its proposed structure is shown in Figure 5). 

This complex exhibited high RCP (>90%), good stability in vitro, and a high binding affinity to hydroxyapatite (97.02%). ^99m^TcN-ALNDTC was hydrophilic (log *P* = −1.51) and showed weak binding to human serum albumin (25.04%). Biodistribution studies in normal mice revealed that the complex showed high bone uptake (28.95% ID/g at 4 h) and good bone-targeting properties, with significantly greater bone accumulation than conventional [^99m^Tc]Tc-MDP. SPECT imaging in rabbits further confirmed its pronounced accumulation in bone. However, the relatively high renal uptake and the less favorable bone-to-blood and bone-to-muscle ratios compared with those of some other analogs may limit its imaging contrast and potential application. In 2018, our group [74] further reported two ^99m^Tc-labeled complexes of pamidronate dithiocarbamate (PAMDTC)—^99m^TcN-PAMDTC and ^99m^TcO-PAMDTC—both of which exhibited high RCP exceeding 97%. (Their proposed structures are shown in Figure 5). Compared with ^99m^TcO-PAMDTC, the ^99m^TcN-PAMDTC complex demonstrated greater hydrophilicity (log *P* = −1.78), lower binding affinity to human serum albumin (10.15%), and a greater binding affinity to hydroxyapatite (93.15%). Biodistribution studies in mice revealed that ^99m^TcN-PAMDTC achieved greater bone uptake (21.17% ID/g at 4 h) and significantly superior bone-to-blood and bone-to-muscle ratios than ^99m^TcO-PAMDTC did. SPECT imaging in rabbits further confirmed its pronounced accumulation in bone. The study indicated that the ^99m^TcN core, compared with the ^99m^TcO core, significantly improved the physicochemical and bone-targeting properties of the labeled complexes. The results from these two studies indicate that the bone imaging agent developed using alendronate as the targeting agent exhibits slightly superior performance compared with that based on pamidronate, a finding further confirmed in our subsequent research.

**Figure 6:**
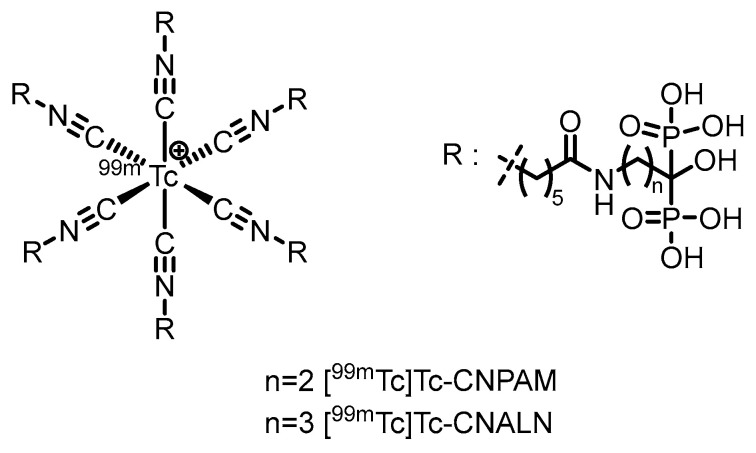
The possible structures of ^99m^Tc-complex-conjugated bisphosphonate compounds: [^99m^Tc]Tc-CNPAM, [^99m^Tc]Tc-CNALN.

In 2022, our group [75] synthesized two isocyanide-containing bisphosphonate derivatives (CNALN and CNPAM) and successfully prepared their corresponding ^99m^Tc-labeled complexes, [^99m^Tc]Tc-CNALN and [^99m^Tc]Tc-CNPAM. (Their proposed structures are shown in Figure 6). This series of complexes exhibited high RCP (>95%), good stability in vitro, hydrophilicity, and a high binding affinity for hydroxyapatite. Biodistribution studies in mice revealed that [^99m^Tc]Tc-CNALN demonstrated greater bone-to-background ratios at 60 min postinjection. SPECT imaging further confirmed its pronounced accumulation in bone, suggesting its potential as a promising bone-targeting agent. However, its bone uptake (11.86% ID/g for [^99m^Tc]Tc-CNALN) was lower than that of the clinically used [^99m^Tc]Tc-MDP, which may limit its sensitivity and application potential in bone imaging.

### 2.2. PET Tracers

As a widely used PET bone imaging agent, [^18^F]NaF benefits from high bone uptake and rapid blood clearance, enabling the acquisition of high-quality images within 30–60 min postinjection [76,77,78,79,80,81]. The production of ^18^F relies on cyclotrons. Although cyclotron production capacity has become increasingly mature worldwide, with favorable cost-effectiveness and accessibility, enabling the supply of stable, high-activity products for centralized distribution networks, the clinical application of ^18^F-labeled tracers remains highly dependent on the availability of local cyclotron facilities. In regions lacking such infrastructure, its widespread adoption continues to face significant constraints.

^68^Ga, a PET nuclide supplied by the ^68^Ge/^68^Ga generator, is highly regarded for its on-demand availability without the need for a cyclotron. Additionally, certain commercial generators have gained approval from regulatory authorities, leading to the widespread global proliferation of [^68^Ge]/[^68^Ga] generators and their production facilities in recent years [82]. Its 68 min half-life is ideal, allowing sufficient time for radiopharmaceutical preparation and imaging while minimizing radiation exposure. Furthermore, the relatively low energy of the positrons emitted by ^68^Ga results in higher spatial resolution in images. Chemically, Ga^3+^ ions can form stable complexes with chelators such as DOTA, enabling the facile construction of diverse targeted imaging agents. This same chemistry underpins the “theranostic” paradigm: after a ^68^Ga-labeled agent is used to diagnose and localize disease, the same targeting molecule labeled with therapeutic radionuclides such as ^90^Y or ^177^Lu can be used for precise treatment, creating a perfect diagnostic-therapeutic closed loop [32,33,36].

**Figure 7:**
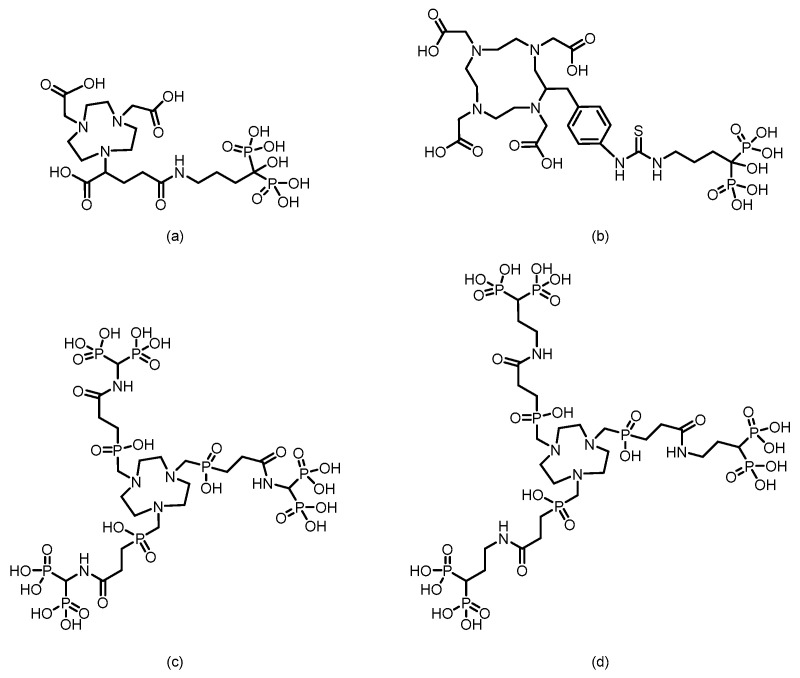
Chemical structures of the bifunctional bisphosphonate chelators: (**a**) NOTA-BP, (**b**) DOTA-Bn-SCN-HBP, (**c**) TRAP(MDP)_3_, (**d**) TRAP(PDP)_3_.

In 2011, Kentaro Suzuki et al. [83] synthesized a ^68^Ga-labeled bisphosphonate conjugate ([^68^Ga]NOTA-BP). (The structures of its corresponding ligand is shown in Figure 7). The compound was radiolabeled by heating for just 10 min, achieving high radiochemical purity (RCP > 95%) and demonstrating good stability in plasma. In hydroxyapatite binding assays, [^68^Ga]NOTA-BP exhibited high affinity (K*d* = 0.0937 μM). In Wistar rats, [^68^Ga]NOTA-BP showed rapid blood clearance and a high bone-to-blood ratio (reaching 1204 at 2 h). While its bone uptake (4.75% ID/g) was lower than that of [^18^F]fluoride, its blood clearance was faster. In a PC-3 bone metastasis mouse model, osteolytic lesions were successfully detected by microPET imaging at 1 h postinjection.

In 2011, Kazuma Ogawa et al. [84] designed and synthesized a gallium-radiolabeled complex-bisphosphonate conjugate (^67^Ga-DOTA-Bn-SCN-HBP). (The structures of its corresponding ligand is shown in Figure 7). This compound was obtained with high radiochemical purity (RCP > 95%) and did not require a purification step. In the hydroxyapatite binding assay, ^67^Ga-DOTA-Bn-SCN-HBP exhibited high affinity, whereas the negative control, ^67^Ga-DOTA, showed no binding. In a biodistribution study in mice, ^67^Ga-DOTA-Bn-SCN-HBP demonstrated significant bone uptake as early as 10 min postinjection (femoral uptake: 17.44% ID/g), which increased steadily to 23.53% ID/g by 3 h, while the radioactivity in nonbone tissues was rapidly cleared. The femur-to-blood ratio reached 245.96 at 3 h. Preinjection of excess alendronate significantly inhibited its bone uptake, indicating that its bone targeting relies on the specific interaction between the bisphosphonate moiety and bone minerals. This study demonstrates that ^67^Ga-DOTA-Bn-SCN-HBP possesses the pharmacokinetic characteristics of an ideal bone imaging agent and provides valuable insights for the molecular design of subsequent ^68^Ga-labeled PET bone imaging agents.

In 2012, Notni et al. [85] synthesized two trimeric bisphosphonate TRAP conjugates (TRAP(MDP)_3_ and TRAP(PDP)_3_) and labeled them with gallium-68 to obtain the corresponding Ga-68-labeled probes. (The structures of their corresponding ligands are shown in Figure 7). These probes were successfully applied for bone microPET imaging in a rat model, demonstrating good bone uptake and a low soft tissue background, which confirmed their suitability for PET bone imaging. However, Ga-71 nuclear magnetic resonance data unexpectedly revealed that Ga(III) was not located within the chelating cavity of TRAP but was instead bound by the bisphosphonate units in the side chains. Although TRAP itself possesses excellent Ga(III) chelating properties, it served merely as a trimeric scaffold in these conjugates, with the actual Ga-68 binding site being the bisphosphonate groups.

**Figure 8:**
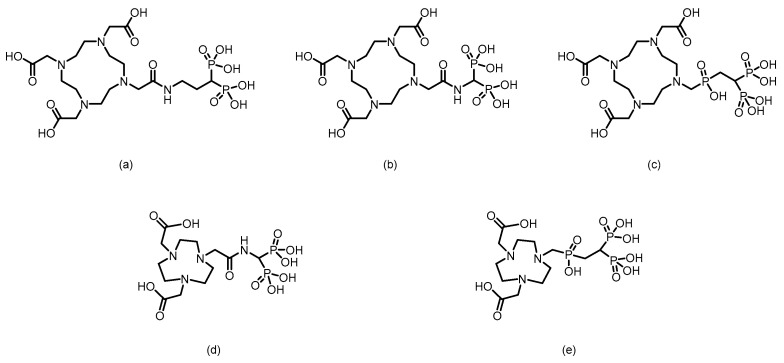
Chemical structures of the bifunctional bisphosphonate chelators: (**a**) BPAPD, (**b**) BPAMD, (**c**) BPPED, (**d**) NOTAM^BP^, and (**e**) NO2AP^BP^.

The F. Rösch team [86] labeled a macrocyclic bisphosphonate-DOTA conjugate with ^68^Ga in 2012, obtaining ^68^Ga-BPAMD with a postpurification RCP exceeding 98%. In vitro, it demonstrated a high binding affinity to hydroxyapatite (81.5%) and exhibited good stability. In healthy rats, the tracer rapidly accumulated in skeletal tissue and was excreted renally. In a rat model of bone metastasis, its uptake in tumor sites was nearly 4 times greater than that in normal bone, enabling clear visualization of small 1–3 mm lesions. Furthermore, its skeletal uptake could be competitively inhibited by other bisphosphonates, confirming the specificity of its activity. In subsequent research, their team [87] used BPAMD as the lead compound and derived two new ligands, BPAPD and BPPED, by modifying the linker between the bisphosphonate group and the DOTA macrocycle or the bisphosphonate side chain. (The structures of their corresponding ligands are shown in Figure 8). After radiolabeling all three with ^68^Ga and comparing their performance, [^68^Ga]BPPED—in which the linker was replaced with a phosphonate ester—demonstrated the best characteristics. It achieved a bone uptake of 2.58 ± 0.33% ID/g in healthy Wistar rats, which was significantly greater than that of [^68^Ga]BPAMD (1.68 ± 0.16% ID/g) and [^68^Ga]BPAPD (2.09 ± 0.15% ID/g). These results confirm that this structural modification strategy effectively enhances bone-targeting performance. In 2014, Holuba et al. [88] applied this strategy to develop two NOTA-based bisphosphonate ligands for ^68^Ga labeling: NOTAM^BP^ (with an acetamide linker) and NO2AP^BP^ (with a methylene phosphonate linker). (The structures are shown in Figure 8). Similarly, [^68^Ga]NO2AP^BP^ showed greater bone uptake than [^68^Ga]NOTAM^BP^ (4.37 ± 0.92 vs. 1.12 ± 0.36% ID/g). Moreover, [^68^Ga]NO2AP^BP^ demonstrated superior bone uptake (SUV = 6.19 ± 1.27 at 60 min in rats), outperforming both conventional tracers such as [^18^F]NaF (SUV = 4.87 ± 0.32) and reported ^68^Ga-DOTA bisphosphonates (e.g., [^68^Ga]BPPED with an SUV of 4.63 ± 0.38). It also showed favorable kinetics: low soft-tissue retention (kidney SUV = 0.26 ± 0.09), rapid renal clearance, and clear bone imaging within 15 min. However, its labeling requires stringent conditions (>60 °C, pH ~ 3) and suffers from slow kinetics due to potential “out-of-cage” intermediate formation.

**Figure 9:**
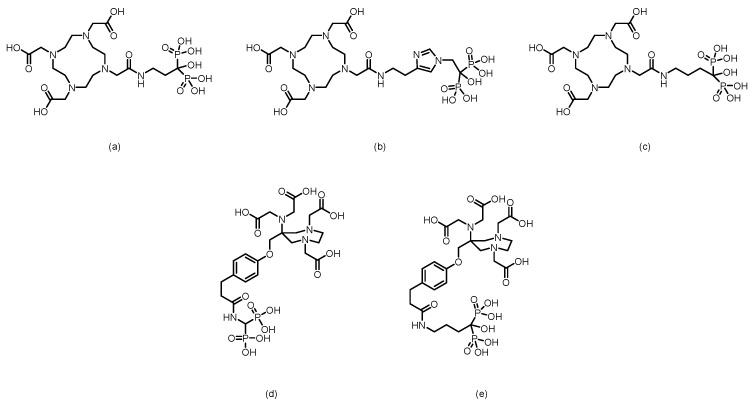
Chemical structures of the bifunctional bisphosphonate chelators: (**a**) DOTA^PAM^, (**b**) DOTA^ZOL^, (**c**) DOTA-ALN, (**d**) PhenA-BPAMD, and (**e**) PhenA-HBP.

In 2016, F. Rösch’s team [89] reported two novel DOTA-α-OH-bisphosphonates (DOTA^PAM^ and DOTA^ZOL^) based on pamidronic acid and zoledronic acid. (The structures are shown in Figure 9). Radiolabeling with ^68^Ga achieved radiochemical yields of 80% to 95% within 15 min, with an RCP exceeding 98% after purification. In in vitro hydroxyapatite adsorption experiments, both [^68^Ga]DOTA^PAM^ and [^68^Ga]DOTA^ZOL^ demonstrated exceptionally high adsorption rates (91.2% and 92.7%, respectively), significantly surpassing the α-H-bisphosphonate control [^68^Ga]BPAPD (83.0%), thus demonstrating their strong binding affinity to bone mineral surfaces. Furthermore, biodistribution studies and small-animal PET imaging in healthy Wistar rats, conducted in comparison with [^18^F]NaF and a known DOTA-α-H-bisphosphonate conjugate (BPAPD), revealed that the tracers exhibited very low uptake in soft tissues, rapid renal clearance, and high accumulation in bone. Among them, [^68^Ga]DOTA^ZOL^ performed the best (femur SUV = 5.4 ± 0.6), followed by [^18^F]NaF (femur SUV = 4.8 ± 0.2), [^68^Ga]DOTA^PAM^ (femur SUV = 4.5 ± 0.2), and [^68^Ga]BPAPD (femur SUV = 3.2 ± 0.3). Moreover, [^68^Ga]DOTA^ZOL^ provided high-contrast PET images, which is consistent with the trends observed in the biodistribution data, highlighting its potential as a promising bone imaging agent.

Fakhari et al. [90] successfully synthesized DOTA-alendronate (DOTA-ALN) and radiolabeled it with ^68^Ga at 92–95 °C for 10 min to obtain ^68^Ga-DOTA-ALN. (The structure of its corresponding ligand is shown in Figure 9). After solid-phase purification, the complex exhibited a radiochemical purity of >99% and a specific activity of 310–320 GBq/mmol. It demonstrated good in vitro stability, remaining stable for up to 90 min, with a partition coefficient log P of −2.91. Maximum ligand binding (65%) was observed in the presence of 50 mg of hydroxyapatite. In biodistribution studies in rats, the complex was primarily excreted via the kidneys, with bone uptake remaining low (<1% ID/g) at 30 min postinjection, while significant uptake was observed in gastrointestinal organs such as the liver, intestine, and colon. Sequential imaging further confirmed the minimal accumulation of the complex in bone tissue.

In 2016, Hank F. Kung’s team [91] developed two bisphosphonate derivatives, PhenA-BPAMD and PhenA-HBP, on the basis of the AAZTA chelator and labeled them with gallium-68 to obtain the corresponding ^68^Ga-labeled probes. (The structures of their corresponding ligands are shown in Figure 9). These probes could be efficiently labeled at room temperature within 5 min (pH 4.0), achieving an RCP of 93–98% without further purification. In healthy CD-1 mice, ^68^Ga-PhenA-BPAMD exhibited excellent bone uptake (11.0% ID/g at 60 min) and high bone-to-blood and bone-to-muscle ratios and demonstrated spine imaging results in rat microPET studies comparable to those of [^18^F]NaF. However, ^68^Ga-PhenA-BPAMD showed poor stability in plasma (the RCP decreased to 46.7% at 120 min), which may lead to slower blood clearance. Additionally, when concentrations of metal ions such as Zn^2+^ and Cu^2+^ exceeded that of the chelating ligand, they significantly inhibited the ^68^Ga labeling efficiency.

**Figure 10:**
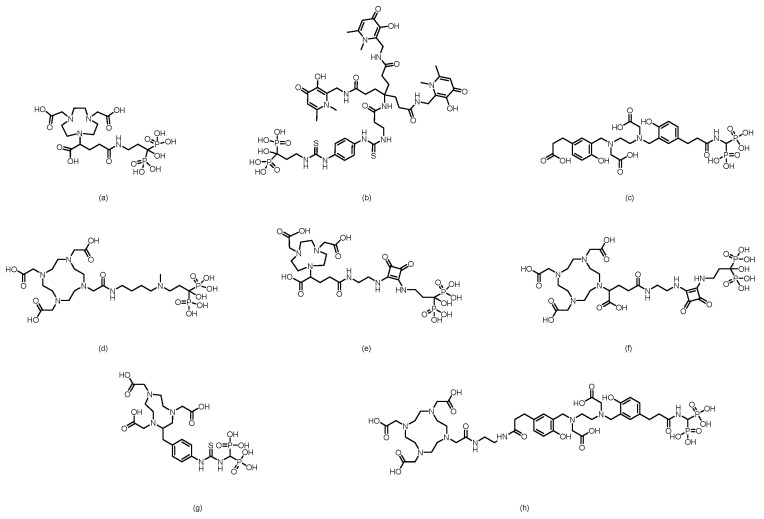
Chemical structures of the bifunctional bisphosphonate chelators: (**a**) NODPAM, (**b**) THP-Pam, (**c**) HBEDCC-BP(P15-041), (**d**) DOTA-IBA(TBM-001), (**e**) NODAGA.SA.PAM, (**f**) DOTAGA.SA.PAM, (**g**) NOTA-conjugated bisphosphonate, and (**h**) P15-073.

In 2020, Ashhar et al. [92] reported the preparation, characterization, and radiolabeling of a novel PET bone imaging agent—[^68^Ga]Ga-NODAGA-pamidronic acid ([^68^Ga]Ga-NODPAM). (The structure of its corresponding ligand is shown in Figure 10). Under conditions of pH 4.5 and 60 °C, an RCP of up to 95% could be achieved within 10–15 min. In in vivo biodistribution studies in healthy Sprague Dawley rats, the tracer showed uptake in bone (femoral %ID/g: 1.84% at 1 h p.i., 1.94% at 2 h p.i.), along with rapid blood clearance and low retention in soft tissues. At 2 h postinjection, the bone-to-blood and bone-to-muscle ratios reached 27.53 and 64.37, respectively.

Keeling et al. [93] developed a novel bisphosphonate PET imaging agent, [^68^Ga]Ga-THP-Pam. (The structure of its corresponding ligand is shown in Figure 10). This compound, which connects a THP chelator to pamidronic acid via a thiourea bond, can be labeled in a single step at room temperature and neutral pH within 5 min, achieving a radiochemical purity greater than 95% without the need for purification. In vitro studies demonstrated that [^68^Ga]Ga-THP-Pam has high affinity for hydroxyapatite and a broad range of other calcium salts relevant to vascular calcification, whereas [^18^F]NaF is selective only for hydroxyapatite. In healthy mouse models, the tracer showed rapid blood clearance (half-life < 10 min) and high uptake in bone tissue, with femoral uptake reaching 14.1 ± 3.7% ID/g at 2 h and a bone-to-muscle ratio of approximately 40. However, the renal uptake of the compound was relatively high (1.4–2.5% ID/g at 2 h).

In 2020, Hank F. Kung’s team [93] developed a ^68^Ga-labeled HBED-CC-bisphosphonate derivative, [^68^Ga]Ga-HBED-CC-BP (also known as [^68^Ga]Ga-P15-041). (The structure of its corresponding ligand is shown in Figure 10). Radiolabeling with ^68^GaCl_3_ in sodium acetate buffer at room temperature for 10 min resulted in >90% RCY and RCP. It demonstrated excellent stability both in vitro and in vivo, along with high binding affinity to hydroxyapatite (92.3%). In biodistribution and microPET imaging studies in mice and rats, [^68^Ga]Ga-HBED-CC-BP showed excellent bone uptake and retention comparable to that of [^18^F]NaF, with bone uptake reaching 23.93%ID/g at 2 h and a maximum bone-to-muscle ratio of 447. It also exhibited rapid blood clearance, significantly reduced uptake in nontargeted organs (e.g., lungs and spleen) within 45 min, and prompt renal excretion without significant accumulation after 2 h. A fully automated synthesis module was developed for the reproducible preparation of clinical doses, positioning this probe as a promising and convenient alternative to [^18^F]NaF that does not require an onsite cyclotron and is suitable for imaging bone disorders and bone metastases.

In 2022, Chen’s team [94] developed a ^68^Ga-labeled bisphosphonate bone imaging probe, [^68^Ga]Ga-DOTA-IBA (also named [^68^Ga]Ga-TBM-001), based on the third-generation bisphosphonate ibandronic acid. (The structure of its corresponding ligand is shown in Figure 10). The probe was synthesized by reacting at 95 °C and pH 4–5 for 15 min, achieving high radiochemical purity (>97%). It exhibited excellent stability both in vitro and in vivo, with high bone targeting specificity. In normal mice, femoral uptake reached 8.365% ID/g at 2 h, accompanied by low uptake in nontarget organs and tissues. In a BALB/c nude mouse PC-3 bone metastasis model, PET/CT imaging at 1.5 h postinjection revealed high tracer uptake in osteolytic lesions of the left tibia, with an SUVmax of 10.3 and a target-to-nontarget (T/NT) ratio of 6.3, enabling clear identification of bone metastatic lesions. Preliminary human imaging studies demonstrated that compared with [^99m^Tc]Tc-MDP SPECT/CT, [^68^Ga]Ga-DOTA-IBA PET/CT detected more lesions, with T/NT ratios ranging from 5.8 to 9.1. The probe showed a favorable safety profile, rapid blood clearance, and predominant renal excretion, highlighting its potential for diagnosing bone metastases and future theranostic applications with ^177^Lu/^225^Ac.

Greifenstein et al. [95] developed two bisphosphonate derivatives based on squaric acid-conjugated pamidronate, NODAGA.SA.PAM and DOTAGA.SA.PAM. (The structures of their corresponding ligands are shown in Figure 10). Both ligands were efficiently labeled with ^68^Ga by reacting in sodium acetate buffer (pH 4.5) at 95 °C for 15 min. While [^68^Ga]Ga-NODAGA.SA.PAM achieved a quantitative radiochemical yield even with as little as 5 nmol of the precursor, [^68^Ga]Ga-DOTAGA.SA.PAM reached a radiochemical yield of up to 90%. Both probes demonstrated good in vitro stability (>95% over 2 h). In healthy Wistar rats, [^68^Ga]Ga-NODAGA.SA.PAM showed rapid blood clearance and significant bone uptake, with the highest accumulation in the metabolically active epiphyseal regions (SUV = 22.9). It was primarily excreted renally and achieved an exceptionally high epiphysis-to-blood ratio of 299.1.

In 2024, Chakraborty et al. [96] developed a ^68^Ga-labeled NOTA-conjugated bisphosphonate probe, [^68^Ga]Ga-3. (The structure of its corresponding ligand is shown in Figure 10). This probe can be efficiently radiolabeled at room temperature and a pH of 3.5 within 15 min, achieving an RCP greater than 98%. It demonstrated excellent in vitro stability (>95% over 4 h) in both physiological saline and human serum, along with high affinity for hydroxyapatite (K*d* = 907 ± 14 mL/g). In heathy Wistar rats, the probe resulted in rapid and significant femoral accumulation (2.48 ± 0.18% ID/g at 60 min) and was cleared quickly via the renal route, resulting in a high femur-to-blood ratio of 121.7 at 60 min. PET imaging in prostate cancer patients confirmed that [^68^Ga]Ga-3 effectively detected small bone metastatic lesions, with lesions showing an SUV 13–16 times that of normal bone. However, the radiolabeling condition at pH 3.5 requires dilution and pH adjustment prior to injection, which may pose certain limitations for its widespread clinical adoption.

In 2024, Hank F. Kung’s team [97] further modified [^68^Ga]Ga-P15-041 by conjugating an additional DOTA ring, resulting in the ligand P15-073. (The structure of its corresponding ligand is shown in Figure 10). This ligand features both DOTA and HBED-CC chelating groups, enabling a theranostic strategy in which HBED-CC selectively binds ^68^Ga for diagnosis and DOTA chelates ^177^Lu for therapy, forming the matched pair [^68^Ga]Ga-P15-073 and [^177^Lu]Lu-P15-073. Radiolabeling of [^68^Ga]Ga-P15-073 was efficiently accomplished at room temperature within 10 min, achieving an RCP exceeding 95%. In vitro hydroxyapatite binding of the tracer reached 96.7%. In normal mice, the tracer was rapidly taken up by bone, reaching a peak uptake (12.1% ID/g) at 30 min postinjection, while it was rapidly cleared from nontarget organs. In an A549 bone metastasis mouse model, PET/CT imaging with [^68^Ga]Ga-P15-073 clearly distinguished and visualized bone destruction lesions, with uptake in the metastatic foci reaching 4 times that in the normal tibia at 60 min postinjection.

**Figure 11:**
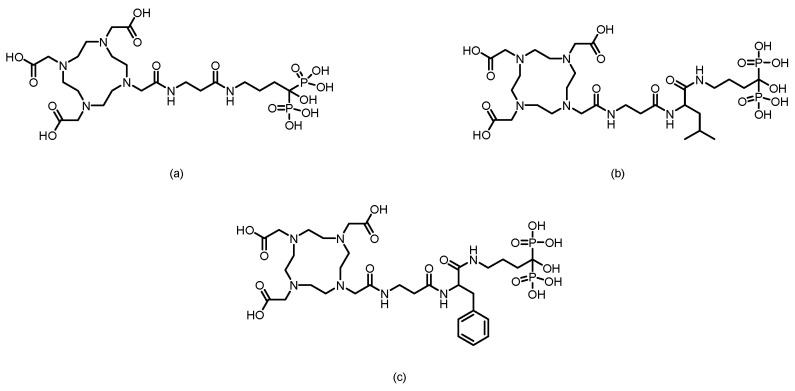
Chemical structures of the bifunctional bisphosphonate chelators: (**a**) AABP1, (**b**) AABP2, (**c**) AABP3.

In 2025, Lin et al. [98] utilized alendronic acid as the bone-targeting core. By modifying it with different amino acid linkers (β-alanine, leucine + β-alanine, phenylalanine + β-alanine) conjugated to the DOTA chelator, they successfully developed three ^68^Ga-labeled PET tracers for bone metastasis diagnosis: [^68^Ga]Ga-AABP1, [^68^Ga]Ga-AABP2, and [^68^Ga]Ga-AABP3. (The structures of their corresponding ligands are shown in Figure 11). All three compounds exhibited high radiochemical purity (RCP >98%) and high molar activity (~7.4 GBq/μmol), as well as excellent stability both in vitro and in vivo. In normal BALB/c mice, [^68^Ga]Ga-AABP3 demonstrated the best performance, achieving a bone uptake of 29.34 ± 1.88% ID/g at 1.5 h postinjection. It showed low uptake in nontargeted organs (liver, lung, and muscle) with rapid clearance, resulting in high bone-to-kidney and bone-to-muscle ratios of 20.54 ± 1.02 and 107.95 ± 7.03, respectively. In an A549 bone metastasis mouse model, metastatic lesions were clearly visualized, with a significantly greater SUVmax in the foci (2.67 ± 0.10) than in the contralateral normal bone at 1.5 h postinjection, and the tumor-to-muscle ratio reached 6.25 ± 0.61.

## 3. Bisphosphonate-Based Targeted Therapeutics for Bone Metastases

The clinical application of radionuclides is informed by their distinct physical properties, such as half-life, radiation type, and energy. In the field of targeted therapy for bone metastases, this is reflected primarily in the classification of therapeutic radionuclides on the basis of the type of particles they emit, namely, α-emitters, β-emitters, and Auger electron emitters. These three types of radiation differ significantly in their physical characteristics: alpha particles are characterized by high energy and a short range, whereas beta particles possess moderate energy and range, and Auger electrons are defined by their low energy and extremely limited action radius [99,100,101,102,103,104]. Consequently, these distinct properties guide the clinical development of tailored, precise treatment strategies for individual patients.

### 3.1. β-Emitter Radiopharmaceuticals

^177^Lu, a therapeutic radionuclide produced in nuclear reactors, is highly regarded for its exceptional efficacy in targeted radionuclide therapy. Its physical half-life of approximately 6.7 days provides a sufficient time window for drug quality control, distribution, and the implementation of multidose treatment regimens. In terms of radiation characteristics, the beta particles emitted by ^177^Lu have moderate energy, enabling effective eradication of tumor tissue at a microscopic scale, while their limited penetration depth serves as a natural physical barrier for protecting healthy tissues. Most importantly, its high flexibility in terms of coordination chemistry allows the Lu^3+^ ion to form exceptionally stable complexes with various macrocyclic chelators, notably DOTA. This capability facilitates the development of a versatile platform for creating radiopharmaceuticals that target different disease sites.

**Figure 12:**
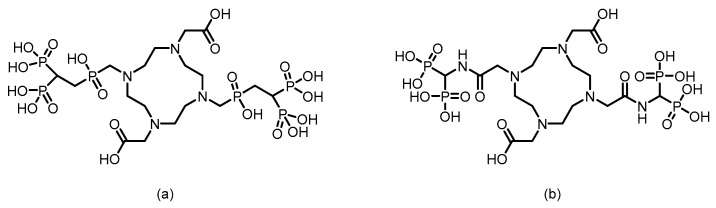
Chemical structures of the bifunctional bisphosphonate chelators: (**a**) DO2A(P^BP^)_2_, (**b**) DOTA(M^BP^)_2_.

In 2016, F. Rösch’s team [105] reported six macrocyclic bisphosphonate ligands (including monomeric and dimeric structures) based on DOTA, DO2A, and NO2A labeled with ^177^Lu, achieving an RCY of more than 98%. (The structures of their corresponding ligands are shown in Figure 12). Experiments in healthy rat models revealed that all ^177^Lu-bisphosphonate complexes exhibited bone-specific accumulation characteristics. Among them, the dimeric [^177^Lu]Lu-DO2A(P^BP^)_2_ and [^177^Lu]Lu-DOTA(M^BP^)_2_ complexes demonstrated high bone uptake but slow blood clearance. In contrast, the monomeric complex [^177^Lu]Lu-BPAMD (the ligand structure is shown in Figure 8) exhibited superior bone-to-soft tissue ratios. One hour postinjection, the SUV of [^177^Lu]Lu-BPAMD in the femur was 4.84 ± 0.44, with a bone-to-blood ratio of 135.6 and a bone-to-muscle ratio of 251.7. SPECT/CT imaging revealed high contrast.

**Figure 13:**
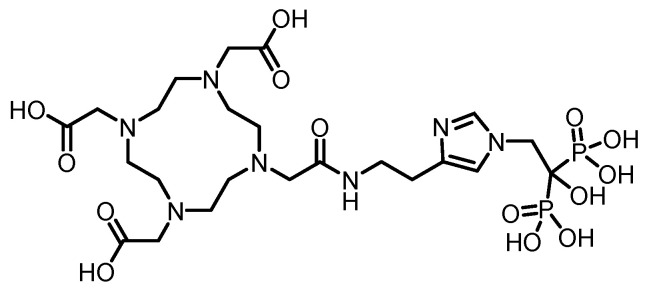
Chemical structure of DOTA^ZOL^.

In 2016, F. Rösch’s team [89] reported the DOTA-conjugated bisphosphonate ligand DOTA^ZOL^ (the ligand structure is shown in Figure 13) and labeled it with ^177^Lu to obtain [^177^Lu]DOTA^ZOL^. The labeling process achieved quantitative incorporation of the radionuclide after 30 min of reaction at 98 °C, with an RCY exceeding 98%. In ex vivo biodistribution studies in healthy Wistar rats, [^177^Lu]DOTA^ZOL^ exhibited rapid and high bone uptake with minimal accumulation in soft tissues: femoral uptake reached 3.43 ± 0.41 %ID/g at 1 h, whereas blood and muscle levels were only 0.07 ± 0.01 %ID/g and 0.02 ± 0.00 %ID/g, respectively. Its skeletal retention was comparable to that of [^68^Ga]DOTA^ZOL^, and the bone activity remained stable for up to 8 days, indicating a long biological half-life in the target tissue. Small-animal SPECT imaging revealed that [^177^Lu]DOTA^ZOL^ had a favorable target-to-soft-tissue ratio and accumulated markedly in bone, especially in highly metabolic regions such as the epiphyseal plates and joints. However, the renal uptake of [^177^Lu]DOTA^ZOL^ was greater than that of the ^68^Ga-labeled analog (1.78 ± 0.13 %ID/g at 1 h).

**Figure 14:**
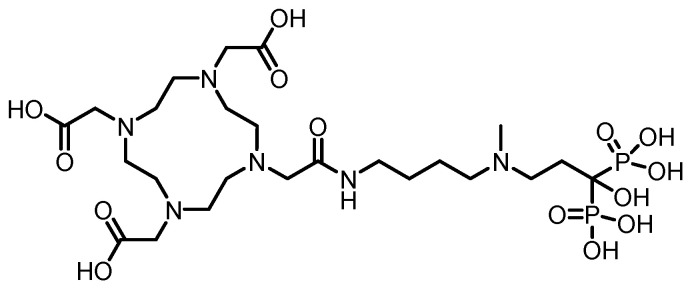
Chemical structure of DOTA-IBA(TBM-001).

In 2023, Chen’s team [106] successfully labeled DOTA-IBA (the ligand structure is shown in Figure 14) with ^177^Lu to obtain ^177^Lu-DOTA-IBA (also known as ^177^Lu-TBM-001). After purification, the RCP exceeded 98%. The compound demonstrated good in vitro stability (>90% over 72 h) and good hydrophilicity (log *P* = −2.29). In mouse models, ^177^Lu-DOTA-IBA exhibited rapid blood clearance, high bone tissue affinity, and high bone-to-liver and bone-to-muscle uptake ratios (68.9 and 203.8 at 72 h, respectively), with excretion occurring primarily via the urinary system. In a preliminary clinical study, five patients with bone metastases received doses ranging from 740 to 1110 MBq. Three of them experienced significant pain relief within three days, with the longest analgesic effect lasting over two months, and no significant toxic side effects were observed. SPECT/CT imaging revealed specific enrichment of the agent in bone metastatic lesions (T/NT ratio up to 22) and minimal uptake in soft tissues.

**Figure 15:**
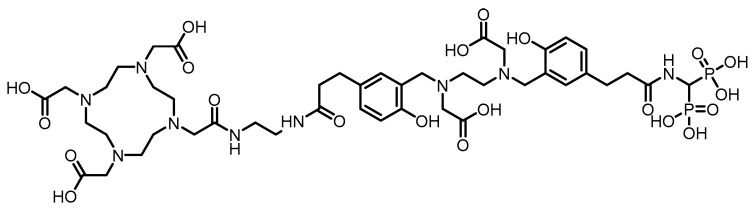
Chemical structure of P15-073.

In 2024, Hank F. Kung’s team [97] successfully labeled P15-073 (the ligand structure is shown in Figure 15) with ^177^Lu to obtain [^177^Lu]Lu-P15-073. ^177^Lu chelated with the DOTA moiety, and after purification, the RCP exceeded 95%. The compound demonstrated a hydroxyapatite binding rate of 90.9 ± 1.1% and maintained good in vitro stability with no significant decomposition after 6 days of incubation in PBS. In vivo distribution studies in normal mice revealed rapid blood clearance and significant bone targeting. Bone uptake reached 12.4% ID/g at 0.5 h postinjection and remained sustained at 11.6% ID/g for up to 24 h, with minimal uptake in other major organs. SPECT/CT imaging confirmed its retention in bone tissue for up to 72 h. Human dosimetry estimates, on the basis of murine biodistribution data, revealed a radiation dose of 0.091 mSv/MBq to osteogenic cells and 0.017 mSv/MBq to red marrow cells, with an effective dose of 0.0245 mSv/MBq.

^186^Re is a therapeutic radionuclide produced through the ^185^Re(n,γ) nuclear reactor irradiation route. Its half-life of approximately 90.6 h (3.8 days) provides ample time for radiopharmaceutical synthesis, quality control, and distribution while also allowing sufficient time for the agent to accumulate at the target site and exert a sustained therapeutic effect. Crucially, it avoids excessive long-term retention in the body. In terms of decay characteristics, ^186^Re emits beta particles with moderate energy, enabling effective destruction of tumor tissue. More importantly, it simultaneously emits characteristic gamma rays with an energy of 137 keV, which can be directly utilized for SPECT imaging. This allows real-time visualization of drug distribution and dosimetric assessment during the treatment course. Furthermore, its chemical behavior is highly similar to that of widely used ^99m^Tc, making it possible to rapidly develop a variety of targeted therapeutic agents on the basis of existing technological platforms.

^186^Re-HEDP is among the earliest approved and widely used radiotherapeutic agents for bone metastases [107,108,109,110]. Owing to its nuclear properties, it offers patients a “gentle yet sustained” analgesic effect. Its extensive clinical data remain a critical benchmark for evaluating the efficacy and safety of any novel bone-targeting radiopharmaceutical.

In 2001, Kothari et al. [111] designed and synthesized a novel cyclic tetraphosphonate ligand, CTMP, and successfully prepared the corresponding ^186^Re-CTMP. Under optimized labeling conditions (pH 2, heating at 100 °C for 30 min, ligand concentration of 9 × 10^−2^ mol/L, and 2 mg of stannous chloride), the compound was obtained with high RCP > 98% and demonstrated good in vitro stability, with no significant decomposition observed after 6 days at room temperature. In biodistribution studies in male Wistar rats, ^186^Re-CTMP exhibited significant bone targeting, with femoral uptake reaching 1.8% ID/g at 3 h postinjection, which was sustained for up to 48 h, while uptake in blood and other soft tissues was minimal. The compound was primarily cleared renally. In imaging studies using rabbit models, scintigraphic images acquired with a dual-head gamma camera were superior to those obtained with ^186^Re-HEDP prepared under the same conditions, and no significant radioactive uptake was observed in other vital organs.

**Figure 16:**
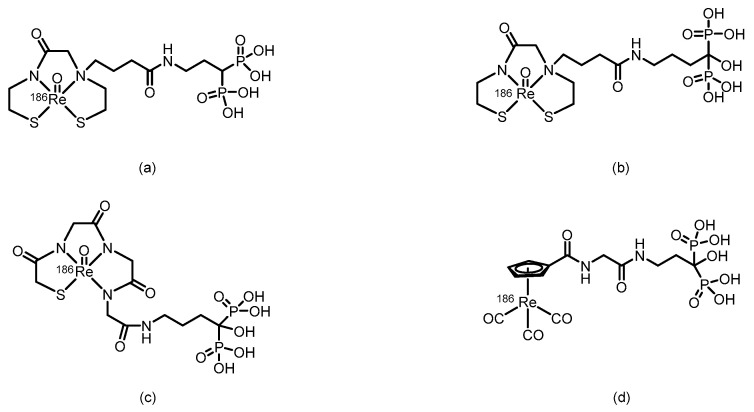
The possible structures of ^186^Re-complex-conjugated bisphosphonate compounds: (**a**) ^186^Re-MAMA-BP, (**b**) ^186^Re-MAMA-HBP, (**c**) ^186^Re-MAG3-HBP, and (**d**)^186^Re-CpTR-Gly-APD.

In 2004, Ogawa et al. [112] developed a ^186^Re-labeled bisphosphonate compound, ^186^Re-MAMA-BP, on the basis of the concept of bifunctional radiopharmaceuticals, using MAMA as the chelator. (Its proposed structure is shown in Figure 16). The compound was prepared by conjugating the stable ^186^Re-MAMA chelate with a bisphosphonate derivative, followed by purification to achieve high RCP (>95%) and an RCY of 32.0 ± 4.1%. In vitro stability tests revealed that after 24 h of incubation in pH 7.0 buffer solution, the intact percentage of ^186^Re-MAMA-BP (81.8 ± 1.7%) was significantly greater than that of the clinical reference drug ^186^Re-HEDP (24.8 ± 0.2%). However, no in vivo biological evaluation was conducted. In 2006, building upon previous work, Ogawa et al. [113] replaced the targeting moiety with alendronate and designed and synthesized ^186^Re-MAMA-HBP. (Its proposed structure is shown in Figure 16). The compound had an RCY of 54%, and after purification, its RCP exceeded 95%. Compared with the previously reported nonhydroxyl derivative ^186^Re-MAMA-BP and the clinical reference drug ^186^Re-HEDP, ^186^Re-MAMA-HBP demonstrated significantly greater affinity for hydroxyapatite in vitro. In mice, it resulted in greater femoral accumulation, faster blood clearance, and lower gastric radioactive uptake, resulting in a higher femur-to-blood radioactivity ratio. These results indicate that introducing a hydroxyl group at the central carbon of the bisphosphonate structure can effectively increase the bone-targeting and accumulation capacity of ^186^Re-chelated bisphosphonate conjugates.

In 2005, Ogawa et al. [114], on the basis of the concept of bifunctional radiopharmaceuticals, designed and synthesized a ^186^Re-labeled MAG3-conjugated bisphosphonate compound, ^186^Re-MAG3-HBP. (Its proposed structure is shown in Figure 16). After purification by HPLC, the RCP of the compound exceeded 95%. In vitro stability experiments demonstrated that no significant decomposition occurred after 24 h of incubation in phosphate buffer, which was far superior to the 30% remaining for ^186^Re-HEDP. Biodistribution studies in normal ddY mice revealed that ^186^Re-MAG3-HBP exhibited rapid and sustained bone targeting, with femoral uptake reaching 27.19% ID/g as early as 30 min postinjection and remaining at 24.51% ID/g after 24 h, which was significantly greater than that of ^186^Re-HEDP. Moreover, compared with that of ^186^Re-HEDP, blood clearance of ^186^Re-MAG3-HBP was faster, and gastric radioactive accumulation was significantly lower.

In 2007, Uehara et al. [107] reported a ^186^Re-labeled bisphosphonate conjugate, ^186^Re-CpTR-Gly-APD, using cyclopentadienylrhenium tricarbonyl as the radiometal chelator. (Its proposed structure is shown in Figure 16). After purification, the resulting compound exhibited high RCP (>95%) and a relatively high RCY (25%). Compared with the clinically used ^186^Re-HEDP, ^186^Re-CpTR-Gly-APD demonstrated superior plasma stability, lower plasma protein binding, higher hydroxyapatite-binding affinity, increased bone accumulation, and faster blood clearance in mice, resulting in a higher bone-to-blood radioactivity ratio. However, in HEDP pretreatment experiments, bone uptake remained unaffected, whereas delayed blood clearance and elevated renal radioactivity accumulation were observed. The authors attributed these results to the fact that the HEDP dose used in the experiment did not fully occupy bisphosphonate-binding sites in murine bones, whereas HEDP competitively inhibited the renal bisphosphonate transporter system.

^188^Re, a highly promising therapeutic radionuclide in its own right, can be conveniently obtained via a tungsten-188/rhenium-188 generator system. Its half-life of approximately 17 h strikes a critical balance: while it necessitates efficient onsite preparation and rapid administration, it also ensures that the radioactivity does not persist unduly in the body after treatment, thereby reducing the overall radiation burden on the patient. In terms of decay properties, ^188^Re emits high-energy β particles with relatively deep tissue penetration, enabling the effective treatment of larger tumor lesions. Moreover, it emits characteristic gamma rays at 155 keV, allowing direct SPECT imaging during therapy. This capability facilitates real-time monitoring of the drug’s biodistribution and targeted accumulation, making precise, patient-specific dosimetric assessments possible. Most importantly, akin to ^186^Re, the chemical behavior of ^188^Re closely resembles that of the cornerstone diagnostic radionuclide in nuclear medicine, ^99m^Tc. These findings suggest that the established ^99m^Tc labeling methodologies and targeting ligand platforms can potentially serve as a reference for the development of targeted therapeutic agents based on ^188^Re. Coupled with the generator production model, this similarity enables clinical on-demand availability comparable to that of ^99m^Tc [115,116].

^188^Re-HEDP is a classic bone-targeting radiopharmaceutical used clinically to relieve severe pain caused by bone metastases from various cancers [117,118,119]. Compared with ^186^Re-HEDP, ^188^Re-HEDP has garnered significant attention for clinical promotion because of its convenient on-site availability via elution from a ^188^W/^188^Re generator and its notable cost-effectiveness. Its labeling method is well established and reliable—typically, a one-step complexation of ^188^Re with the HEDP ligand can be achieved under heating conditions (boiling for 10–15 min). Following this straightforward direct-labeling strategy, researchers have developed numerous other agents, such as ^188^Re-labeled alendronate, ibandronate, zoledronate, and similar compounds [120,121,122,123,124,125,126,127].

**Figure 17:**
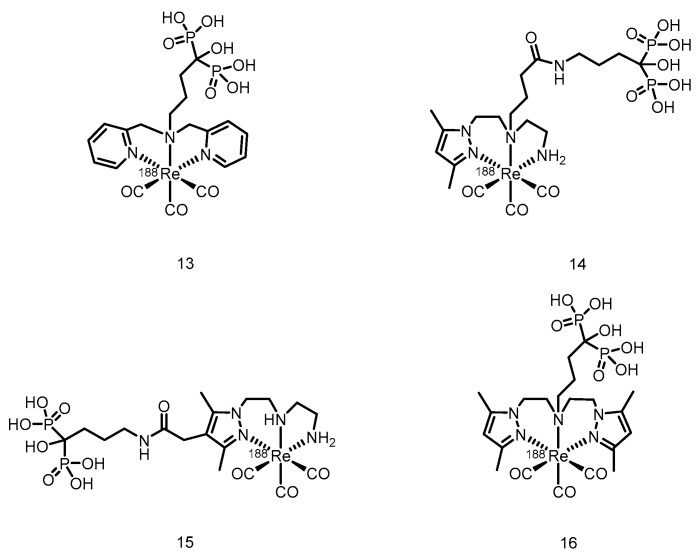
The possible structures of tricarbonyl rhenium-based phosphate-targeting complexes.

In 2010, Torres Martín de Rosales et al. [128] developed a ^188^Re-labeled bisphosphonate conjugate, ^188^Re(CO)_3_-dipicolylamine-alendronate (**13**). (Its proposed structure is shown in Figure 17). The compound was synthesized using a kit-based two-step method, achieving high specific activity (18.8 GBq/mg) and an extremely high radiochemical yield (≥96%), resulting in the formation of a well-defined, stable single species. Compared with the clinically used ^188^Re-HEDP, ^188^Re(CO)_3_-DPA-alendronate (**13**) exhibited superior oxidative stability and very low plasma protein binding in vitro. In a female BALB/c mouse model, higher bone-targeted accumulation (21.2 ± 6.6% ID/g in the femur at 48 h), prolonged retention in bone, and lower thyroid uptake were detected. SPECT/CT imaging and biodistribution studies confirmed that the compound effectively targeted and accumulated in areas of high metabolic bone activity, with minimal soft-tissue uptake.

In 2015, Fernandes et al. [129] labeled a previously developed series of bisphosphonate conjugates with tricarbonyl ^188^Re, obtaining compounds **14** to **16**. (Their proposed structures are shown in Figure 17). After optimized synthesis, compound **15** exhibited an RCP exceeding 95%, high specific activity, and an RCY > 85%. This compound demonstrated high stability both in vitro (in PBS and culture medium) and in vivo, along with a high affinity for hydroxyapatite. In BALB/c mice, compound **15** showed rapid blood clearance, high bone uptake (16.1 ± 3.3% IA/g at 1 h postinjection), and high bone-to-blood and bone-to-muscle radioactivity ratios, indicating its ability to deliver radiation to bone tissue in a highly selective manner. In the metastatic breast cancer cell line MDA-MB-231, compound **15** induced a concentration-dependent radiocytotoxic effect, which was significantly stronger than that of an equivalent concentration of [^188^ReO_4_]^−^, and its ability to induce DNA damage was confirmed via the micronucleus assay. However, some radioactive uptake was observed in the liver (6.9 ± 0.8% IA/g at 1 h postinjection).

^90^Y is an important therapeutic radionuclide produced either through the neutron activation of ^89^Y via the (n,γ) reaction in nuclear reactors or via elution from a ^90^Sr-^90^Y generator. It has a half-life of approximately 64.1 h (2.67 days). In terms of decay characteristics, ^90^Y emits high-energy beta particles (with a maximum energy of 2.28 MeV), which effectively destroy tumor tissue. Moreover, its stable +3 oxidation state and versatile coordination chemistry enable stable labeling of monoclonal antibodies, microspheres, and various targeting molecules.

In 2009, Ogawa et al. [130] conjugated the stable chelator DOTA with alendronate to obtain the ligand DOTA-HBP, which was then labeled with ^90^Y to produce ^90^Y-DOTA-HBP. After purification, the RCP of this compound exceeded 95%, and it remained nearly intact after 1 h of incubation in plasma. Compared with traditional [^90^Y]citrate, [^90^Y]DOTA-HBP resulted in faster blood clearance and lower retention in soft tissues, thereby significantly reducing the radiation dose to nontarget organs. However, its long-term stability in plasma was limited, as it gradually decomposed over time.

**Figure 18:**
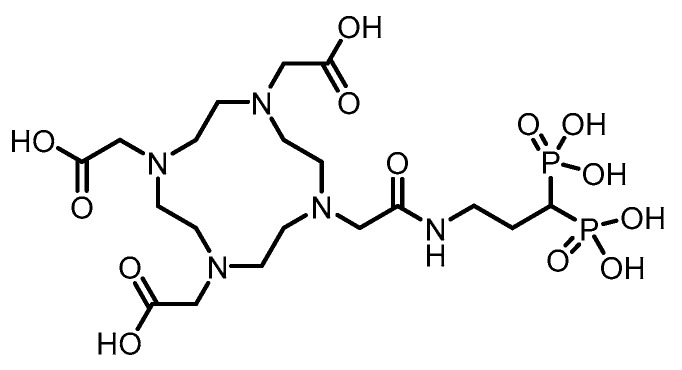
Chemical structure of PBAMD.

In 2016, Rabiei et al. [131] successfully labeled BPAMD (the ligand structure is shown in Figure 18) with ^90^Y, obtaining ^90^Y-BPAMD. Under optimized conditions, the labeled product achieved an RCP greater than 98% and a high specific activity of 3.52 TBq/mmol, demonstrating excellent in vitro stability by remaining intact after 48 h at room temperature and in human serum. In a Syrian rat model, ^90^Y-BPAMD exhibited rapid blood clearance and highly selective uptake in bone tissue, with negligible accumulation in nontarget organs. Through comparative literature analysis, the authors suggested that compared with ^90^Y-citrate, ^90^Y-BPAMD showed superior bone-to-liver and bone-to-lung uptake ratios at 1 h postinjection, and these ratios were comparable to those of ^90^Y-DOTA-HBP, indicating potential advantages in terms of in vivo stability and targeting selectivity. However, as the comparative studies were based on different animal models (rats vs. mice), these findings require further validation through direct comparative studies under identical experimental conditions.

^131^I is an extremely important therapeutic and diagnostic radionuclide. It is produced mainly by the neutron irradiation of stable ^130^Te in nuclear reactors, generating ^131^Te, which then undergoes β^−^ decay, or is extracted from the fission products of highly enriched uranium fuel. Its half-life is approximately 64.0 h (2.67 days). In terms of decay characteristics, ^131^I primarily emits medium-energy β^-^particles (with a maximum energy of 0.606 MeV and an average energy of approximately 0.192 MeV). The short range of these particles in tissues (approximately 1–2 mm) enables precise destruction of lesional tissues. More importantly, ^131^I emits characteristic γ-rays with an energy of 364 keV during decay, which allows it to serve a dual function in both therapy and imaging diagnosis.

**Figure 19:**
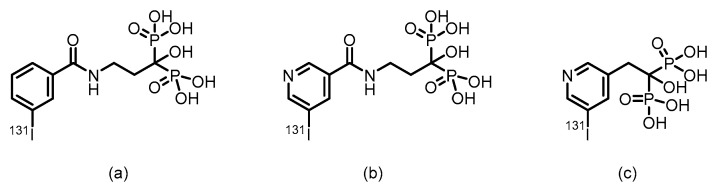
Chemical structures of iodine-labeled compounds: (**a**) ^131^I-IBPB, (**b**) ^131^I-IPPB, (**c**) ^131^I-risedronate.

In 1999, Larsen et al. [132] utilized alendronate as a bone-targeting carrier and successfully synthesized two bisphosphonate derivatives, ^131^I-IBPB and ^131^I-IPPB, by labeling ^131^I via benzoyl and pyridine carbonyl structures, respectively. (Their structures are shown in Figure 19).The labeling yield exceeded 80%, and the compounds demonstrated favorable in vitro and in vivo stability. Biodistribution studies in normal Balb/c mice showed that both compounds exhibited good bone targeting and sustained bone retention. Specifically, ^131^I-IBPB achieved a femoral uptake of 18.22% ID/g as early as 30 min post-injection, peaked at 34.23 ± 7.35% ID/g, and remained at a stable level after 24 h. Additionally, the compound demonstrated rapid blood clearance. However, the estimated bone surface dose of this ^131^I-labeled compound was only about one-third of that of its ^211^At-labeled counterpart, a difference that may be attributed to the longer tissue penetration range of beta particles compared to alpha particles.

In 2025, Lin et al. [133] designed and synthesized ^131^I-labeled bone-targeting agent, ^131^I-risedronate. (Its structure is shown in Figure 19.) The compound attaches ^131^I to the 5-position of the pyridine ring of risedronate via a stable covalent bond, achieving an RCP greater than 99% and an RCY of 80–85%, while also demonstrating good in vitro stability in human plasma (91.3% remained intact after 24 h). In normal BALB/c mice, ^131^I-risedronate exhibited excellent bone-targeting properties, with peak femoral uptake (18.26% ID/g) at 4 h postinjection and sustained retention until day 3 (13.80% ID/g). It also resulted in rapid blood clearance and minimal uptake in nontarget organs. SPECT/CT imaging further confirmed its specific accumulation in bone, with no significant uptake observed in the thyroid region. However, high kidney uptake (9.37% ID/g) was noted at 4 h postinjection, raising potential concerns about renal toxicity that require further evaluation.

Additionally, ^47^Sc is also a highly promising theranostic radionuclide. With a half-life of 3.3 days, it emits both therapeutic β^−^ particles (average energy 162 keV) and γ-rays suitable for SPECT imaging (159.4 keV). In 2017, Leila Deilami-nezhad et al. [134] used the chemically similar ^46^Sc as a surrogate for ^47^Sc to successfully radiolabel the bisphosphonate drug HEDP, resulting in ^46^Sc-HEDP. This probe demonstrated excellent bone targeting, rapid blood clearance, and low non-target organ uptake in animal studies, preliminarily validating the feasibility and potential of scandium-labeled bisphosphonate-based bone-targeting agents.

### 3.2. α-Emitter Radiopharmaceuticals

^211^At is an alpha-emitting therapeutic radionuclide primarily produced via cyclotron irradiation. With a half-life of approximately 7.214 h, it offers a suitable time window for both radiopharmaceutical preparation and clinical administration. In terms of decay characteristics, it undergoes alpha decay (41.8%) emitting high-energy alpha particles at 5.87 MeV, accompanied by Auger electron emission from electron capture (58.2%). Although the associated characteristic X-rays in the 77–92 keV range allow for preliminary SPECT imaging, its widespread application remains constrained by challenges such as limited global cyclotron production capacity and suboptimal in vivo stability.

**Figure 20:**
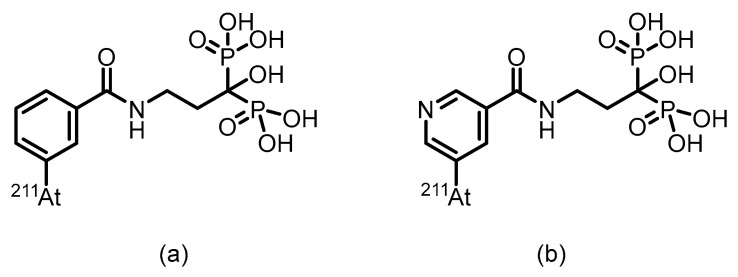
Chemical structures of (**a**) ^211^At-ABPB, (**b**) ^211^At-APPB.

In 1999, Larsen et al. [132] employed alendronate as a bone-targeting carrier and successfully synthesized two bisphosphonate derivatives, ^211^At-ABPB and ^211^At-APPB, by introducing ^211^At via benzoyl and pyridine carbonyl structures, respectively. (Their chemical structures are shown in Figure 20). The labeling yield exceeded 80%, and the compounds demonstrated good in vitro stability. Biodistribution studies in normal Balb/c mice revealed that both compounds exhibited significant bone targeting and sustained bone retention. Specifically, ^211^At-ABPB achieved femoral uptake of 16.63% ID/g as early as 30 min post-injection, peaked at 34.94 ± 8.21% ID/g at 2 h, and remained at 23.88% ID/g after 24 h, indicating stable retention in bone tissue. Furthermore, the compound showed rapid blood clearance, with blood radioactivity falling below 0.9% ID/g within 2 h. Low uptake in the thyroid and stomach further confirmed its high in vivo stability, highlighting its potential for further development as a bone-targeted α-therapeutic agent.

^225^Ac is an alpha-emitting therapeutic radionuclide with a half-life of approximately 10 days. Currently, its production relies mainly on elution from ^229^Th generators, with a global annual output of only a few curies [135,136,137]. The alpha particles emitted during the decay process undergo ultrahigh linear energy transfer (LET ≈ 80 keV/μm), which can directly damage DNA double strands, and a single particle is capable of inducing apoptosis. Although the gamma-ray yield of ^225^Ac is relatively low (similar to the 218 keV photons from ^221^Fr), it still supports preliminary imaging verification during the treatment process.

Recently, researchers have successfully developed the DOTA-chelator-based bisphosphonate-targeting conjugates ^225^Ac-DOTA-IBA and ^225^Ac-DOTA-ZOL and have conducted preliminary evaluations of their potential for treating bone metastases [138,139]. These studies represent a significant step forward for alpha emitters in the field of bone-targeted radiopharmaceutical therapy.

### 3.3. Auger Electron Emitters

^195m^Pt is an Auger-electron-emitting therapeutic radionuclide with a half-life of 4.02 days. It is produced primarily through neutron capture on ^194^Pt, though supply remains limited. Each decay emits an average of approximately 36 Auger electrons, delivering high linear energy transfer (4–26 keV/μm) that enables highly efficient tumor cell killing at the nanoscale. Its accompanying 98.9 keV gamma photons can be used for SPECT imaging, allowing preliminary visualization and monitoring during the therapeutic process.

In 2020, Nadar et al. [140] developed a bone-targeting theranostic agent, ^195m^Pt-BP, by functionalizing the Auger emitter ^195m^Pt with bisphosphonate. The complex demonstrated rapid and persistent accumulation in bone metastases (7.9% ID/g at 1 h, ~75% retained at 7 days), with 7.3-fold higher targeting than ^195m^Pt-cisplatin. It significantly induced DNA damage (4.6-fold increase in γ-H2AX) and apoptosis (3.4-fold increase) in tumor cells without evident nephrotoxicity, providing strong preclinical support for targeted Auger therapy of bone metastases.

^161^Tb is an emerging theranostic radionuclide that is produced primarily by irradiating ^160^Gd targets in high-flux nuclear reactors via the pathway ^160^Gd(n,γ)^161^Gd → β^−^ decay. With a half-life of 6.89 days, it is well suited for clinical radiopharmaceutical preparation and treatment scheduling. In terms of decay characteristics, ^161^Tb undergoes electron capture and emits a large number of Auger electrons [141]. These particles are characterized by highly concentrated energy and an extremely short range (nanometer scale), enabling high-precision tumor cell elimination at the subcellular level. Their nearly negligible tissue penetration also results in the formation of a natural physical barrier that protects surrounding healthy tissues. Additionally, ^161^Tb decay is accompanied by the emission of 48.9 keV and 74.6 keV gamma rays, which can be used for SPECT imaging, thereby integrating therapy and diagnostics. Chemically, the ^161^Tb^3+^ ion exhibits excellent coordination stability and can form stable complexes with macrocyclic chelators such as DOTA. This property allows ^161^Tb to seamlessly integrate into the targeted drug development platforms originally established for ^177^Lu, accelerating its clinical translation [142,143].

Currently, the main challenge facing ^161^Tb lies in the difficulty of large-scale production, with its existing capacity being significantly lower than that of ^177^Lu. Nevertheless, as a representative of “nanoscalpel”-like precision radiotherapy, ^161^Tb demonstrates unique potential in addressing tumor heterogeneity, eradicating micrometastases, and reducing bone marrow toxicity, positioning it as an important developmental direction for the next generation of targeted radionuclide therapy [143].

In 2025, Sitarica et al. [144] reported for the first time the direct radiolabeling of two bisphosphonate ligands—etidronic acid (HEDP) and zoledronic acid (ZOL)—with ^161^Tb for the development of bone-targeting theranostic agents. Labeling was performed under neutral conditions (pH 7), achieving high RCP (>98%) and excellent in vitro stability in both saline and human serum. Both ^161^Tb-bisphosphonate complexes exhibited strong hydrophilicity, high affinity for hydroxyapatite, and moderate-to-high plasma protein binding. Biodistribution studies in healthy Wistar rats demonstrated that both ^161^Tb-HEDP and ^161^Tb-ZOL achieved high and stable bone uptake, rapid blood clearance, and minimal accumulation in soft tissues. ^161^Tb-HEDP showed greater initial bone uptake (9.39 ± 0.30% ID/g), while ^161^Tb-ZOL displayed lower retention in the kidneys and liver, indicating improved safety and selectivity. Compared with unchelated ^161^TbCl_3_, both bisphosphonate complexes resulted in significantly greater bone-to-kidney and bone-to-liver ratios, reflecting superior targeting specificity. Complementary cyclic voltammetry with nonradioactive terbium and density functional theory (DFT) calculations confirmed complex formation, revealing that both phosphonate groups of each ligand coordinate to Tb^3+^. This study demonstrates that ^161^Tb-labeled bisphosphonates, particularly ^161^Tb-ZOL, are promising candidates for the treatment of bone metastases and other skeletal disorders.

In addition, Geeva et al. [145] proposed in 2025 a strategy to construct bipyridyl-bisphosphonate complexes using ^103^Pd and ^109^Pd. These complexes, by releasing Auger electrons and β^−^ particles, demonstrated significantly superior cytotoxicity and DNA-damaging capability against bone-metastatic tumor cells in vitro compared to conventional drugs, providing a new research direction for the treatment of bone metastases.

## 4. Conclusions and Prospects

Bone tissue has long been regarded as one of the most classic and mature targets in radiopharmaceutical research and development because of its high blood perfusion, active bone metabolic turnover, and inherent affinity for hydroxyapatite crystals [146,147,148,149,150]. With the widespread adoption and promotion of nuclear medicine imaging modalities—such as SPECT/CT, PET/CT, and PET/MRI—worldwide, the theranostic strategy based on matched radionuclide pairs has successfully advanced a series of bisphosphonate-based radiopharmaceuticals from the laboratory to clinical application [13,15,37,151,152]. Examples include ^99m^Tc for SPECT imaging paired with ^188^Re for therapy and ^68^Ga for PET imaging paired with ^177^Lu for therapy. Among these, several high-performing ligands have not only achieved a complete closed-loop process of diagnosis guiding therapy and therapy with verified efficacy but have also contributed to the updating and improvement of relevant clinical guidelines through demonstrated clinical benefits [13,15,153,154,155].

During this drug design process, researchers have conducted systematic engineering explorations at the molecular level. By optimizing the bisphosphonate core structure—such as adjusting the side chain length and substituents—bone affinity and metabolic stability are enhanced. Through systematic screening and the adoption of established chelating systems (e.g., DOTA, NOTA, and HYNIC), efficient and stable labeling with different metallic radionuclides is achieved. Furthermore, by meticulously designing the chemical structure of the linker, researchers can modulate the pharmacokinetic behavior of the drug in vivo, ensuring high bone-targeting efficiency while minimizing radiation exposure to nontarget organs. These synergistic design strategies collectively form the chemical foundation for the development of bone-targeted theranostic agents.

Recently, the research frontier in this field has further expanded, with novel radionuclides infusing new vitality into bisphosphonate-based agents. For example, radionuclides such as ^131^I (which emits β-particles for therapy and γ-photons suitable for SPECT imaging) and ^161^Tb (which emits β-particles along with characteristic X-rays/γ-photons applicable to SPECT imaging) have attracted significant attention because of their intrinsic theranostic properties [133,144]. Combining these radionuclides with bisphosphonate carriers can lead to the establishment of a more efficient single-radionuclide theranostic platform. This approach holds promise for simplifying drug preparation and quality-control processes, reducing production costs, and providing key tools for precise dosimetry assessment and real-time treatment response prediction in personalized therapy.

From imaging-assisted therapy to true treatment monitoring and individualized dose adjustment, the system of bisphosphonate-based bone-targeting radiopharmaceuticals continues to evolve. This progression not only aligns with the core tenet of precision medicine—delivering the right dose to the right lesion at the right time—but also directly addresses persistent clinical challenges in the management of bone metastases, such as strong reliance on analgesia, significant side effects of systemic therapies, and limited improvement in patients’ quality of life. Looking ahead, with continued innovation in molecular design strategies, improvements in novel radionuclide supply systems, and the establishment of intelligent dosimetry models, we anticipate that more rationally designed, efficient, accessible, and cost-effective bone-targeting theranostic drugs will emerge. Such advances will truly facilitate the transition from palliative pain relief to curative-intent efficacy, ultimately bringing tangible extensions in survival and dignity to patients.

## Figures and Tables

**Table 1:** Data related to certain Zoledronic acid analogue tracers for bone imaging applications.

Complex	Animal	Time p.i. (h)	Bone Uptake (%ID/g)	Bone/Muscle Ratio	Bone/Blood Ratio	Reference
^99m^Tc-PIDP	Normal mice	2	5.94 ± 0.75	41.54	28.29	[50]
^99m^Tc-EMIDP	Normal mice	1	7.07 ± 0.59	29.79	6.93	[51]
^99m^Tc-i-PIDP	ICR mice	1	9.63 ± 0.64	67.83	20.06	[52]
^99m^Tc-IPrDP	ICR mice	1	11.14 ± 1.85	58.40	6.10	[53]
^99m^Tc-MIPrDP	ICR mice	2	19.6 ± 0.87	196.0	150.8	[54]
^99m^Tc-MIBDP	ICR mice	2	11.2 ± 0.13	140.0	58.9	[54]
^99m^Tc-MIPeDP	ICR mice	2	17.6 ± 0.42	160.0	97.8	[54]
^99m^Tc-DMIDP	ICR mice	1	10.68 ± 0.79	30.67	18.70	[55]
^99m^Tc-IBDP	ICR mice	1	11.4 ± 0.38	51.82	15.20	[56]
^99m^Tc-IPeDP	ICR mice	1	16.2 ± 1.75	51.14	12.97	[56]
^99m^Tc-PIPrDP	ICR mice	1	8.42 ± 0.53	33.68	8.42	[57]
^99m^Tc-PIBDP	ICR mice	1	9.08 ± 0.65	41.27	10.81	[57]
^99m^Tc-PIPeDP	ICR mice	1	10.3 ± 0.61	41.2	20.20	[57]
^99m^Tc-HEIDP	ICR mice	1	6.04 ± 0.80	32.79	-	[58]
^99m^Tc-BIDP	ICR mice	1	22.8 ± 2.32	37.37	-	[59]
^99m^Tc-BIPeDP	ICR mice	1	17.3 ± 0.14	82.38	32.04	[60]
^99m^Tc-EIPrDP	ICR mice	1	13.3 ± 1.23	110.8	38.0	[61]
^99m^Tc-EIBDP	ICR mice	1	11.7 ± 0.28	58.5	22.1	[61]
^99m^Tc-EIPeDP	ICR mice	1	8.69 ± 0.04	37.8	13.8	[61]
^99m^Tc-HBIDP	ICR mice	1	-	-	-	[62]
^99m^Tc-EMIHPBP	Albino mice	1	31.60 ± 0.15	16.90	23.76	[63]

## Data Availability

No new data were created or analyzed in this study. Data sharing is not applicable to this article.

## Abbreviations

The following abbreviations are used in this manuscript:

SPECT	Single Photon Emission Computed Tomography
PET	Positron Emission Tomography
RCP	Radiochemical Purity
RCY	Radiochemical Yield
SUV	standardized uptake value
DFT	density functional theory
